# Sarcoidosis Presenting With Severe Hypercalcemia, Renal Dysfunction, and Bilateral Submandibular Swelling: A Case Report

**DOI:** 10.7759/cureus.114491

**Published:** 2026-08-13

**Authors:** Inês Ferreira, Elsa Araújo, Amanda Rey, Nereida Monteiro, Rafael Lopes Freitas

**Affiliations:** 1 Internal Medicine, Unidade Local de Saúde do Alto Minho, Ponte de Lima, PRT; 2 Palliative Care, Unidade Local de Saúde do Alto Minho, Ponte de Lima, PRT

**Keywords:** acute kidney injury, endobronchial ultrasound, hypercalcemia, sarcoidosis, submandibular gland swelling, transbronchial needle aspiration

## Abstract

Sarcoidosis is a multisystem granulomatous disease that can be complicated by severe hypercalcemia and renal dysfunction. We report the case of a 51-year-old man admitted with severe hypercalcemia, acute kidney injury, weight loss, and bilateral submandibular gland enlargement. The diagnostic workup revealed suppressed parathyroid hormone (PTH), elevated 1,25-dihydroxyvitamin D, hypercalciuria, mediastinal and hilar lymphadenopathy, and pulmonary ground-glass opacities. ^18^F-fluorodeoxyglucose PET/CT (^18^F-FDG PET/CT) showed increased uptake in the submandibular and parotid glands, suggesting inflammatory involvement; however, salivary gland sarcoidosis was not histologically confirmed. Endobronchial ultrasound-guided transbronchial needle aspiration (EBUS-TBNA) demonstrated non-necrotizing granulomas. Together with compatible thoracic imaging and a negative evaluation for alternative granulomatous disorders, this finding supported the diagnosis of pulmonary sarcoidosis. Renal biopsy showed chronic tubulointerstitial nephritis, and although this finding may be compatible with renal involvement in sarcoidosis, direct renal sarcoidosis was not histologically confirmed because of the absence of granulomas. IV fluids and pamidronate led to normalization of serum calcium levels before corticosteroid therapy. After the initiation of prednisolone, renal function and thoracic imaging findings improved, and proteinuria decreased. This case illustrates the importance of considering sarcoidosis in PTH-independent hypercalcemia, particularly when it is associated with renal dysfunction and systemic inflammatory findings, while distinguishing histologically supported disease from possible organ involvement.

## Introduction

Sarcoidosis is a multisystem granulomatous inflammatory disease of unknown etiology, histologically characterized by non-necrotizing granulomas. Although pulmonary and thoracic lymph node involvement predominates, virtually any organ may be affected, leading to a wide spectrum of systemic and often nonspecific symptoms that can pose significant diagnostic challenges [1-3].

The diagnosis relies on a compatible clinical and radiological presentation, histological demonstration of non-necrotizing granulomas in one or more organs, and careful evaluation for alternative causes, particularly infections, malignancy, and other granulomatous disorders [2,3]. However, because of the absence of standardized objective criteria, diagnostic certainty depends on clinicopathological correlation, and atypical presentations may delay recognition [2].

Disorders of calcium metabolism are a recognized but relatively uncommon manifestation of sarcoidosis. Hypercalcemia occurs in approximately 5-10% of patients and is mainly mediated by the extrarenal production of 1,25-dihydroxyvitamin D (calcitriol) by activated macrophages within granulomatous tissue [2,4,5]. Renal dysfunction is often underrecognized and may occur secondary to hypercalcemia and hypercalciuria or result from direct granulomatous interstitial nephritis, occasionally presenting as acute kidney injury [2,6]. Severe hypercalcemia with renal dysfunction may mimic malignancy or primary disorders of calcium metabolism [4-8].

Salivary gland involvement is uncommon in sarcoidosis and most often affects the parotid glands; submandibular manifestations are less frequently reported [9,10]. Clinical swelling or increased ^18^F-fluorodeoxyglucose (^18^F-FDG) uptake may suggest involvement, but neither finding is specific without tissue confirmation.

We report a case of histologically supported pulmonary sarcoidosis presenting with parathyroid hormone (PTH)-independent severe hypercalcemia, acute kidney injury, and bilateral submandibular gland enlargement. Hypercalcemia and renal dysfunction are recognized manifestations of sarcoidosis; the educational value of this case lies in their combination with salivary gland abnormalities, highlighting an atypical initial presentation. In addition, renal biopsy showed chronic tubulointerstitial nephritis without granulomas, requiring careful distinction between confirmed and possible organ involvement.

An abstract of this case was previously presented as a poster at the 17th Meeting of Internal Medicine Residents on June 22, 2024.

## Case presentation

A 51-year-old male carpenter with no relevant medical history and taking no regular medication was admitted for evaluation of progressive bilateral submandibular gland enlargement over the previous month. This was associated with an unintentional weight loss of 26 kg over 10 months, asthenia, and anorexia. He denied dysphagia, fever, night sweats, respiratory symptoms, or calcium/vitamin D supplementation.

Physical examination revealed bilateral, firm, mobile submandibular gland enlargement, with each gland measuring approximately 3 cm. Cardiopulmonary and abdominal examinations were unremarkable. There were no cutaneous lesions or additional palpable cervical or peripheral lymphadenopathy. Laboratory tests showed severe albumin-corrected hypercalcemia of 14.8 mg/dL, acute kidney injury with a serum creatinine level of 2.77 mg/dL (baseline serum creatinine level: 1.01 mg/dL), and normocytic normochromic anemia with a hemoglobin level of 12.8 g/dL. Given the combination of severe hypercalcemia, renal dysfunction, weight loss, and submandibular-region swelling, an extensive diagnostic workup was undertaken.

As shown in Table 1, PTH levels were suppressed, while 25-hydroxyvitamin D levels were not elevated. Further evaluation demonstrated a markedly elevated 1,25-dihydroxyvitamin D level and significant hypercalciuria, supporting the diagnosis of PTH-independent hypercalcemia.

**Table 1:** Etiological investigations. ^18^F-FDG PET/CT: ^18^F-fluorodeoxyglucose positron emission tomography/computed tomography; ACE: Angiotensin-converting enzyme; ANA: Antinuclear antibody; ANCA: Antineutrophil cytoplasmic antibody; anti-CCP: Anti-cyclic citrullinated peptide antibody; anti-dsDNA: Anti-double-stranded deoxyribonucleic acid antibody; anti-Scl-70: Anti-topoisomerase I antibody; anti-SSA/Ro: Anti-Sjögren syndrome-related antigen A/Ro antibody; Anti-SSB/La: anti-Sjögren syndrome-related antigen B/La antibody; BAL: Bronchoalveolar lavage; CD4: Cluster of differentiation 4; CD8: Cluster of differentiation 8; EBUS-TBNA: Endobronchial ultrasound-guided transbronchial needle aspiration; HBV: Hepatitis B virus; HCV: Hepatitis C virus; IGRA: Interferon-gamma release assay; PTH: Parathyroid hormone.

Category	Test	Result	Interpretation
Calcium metabolism	Albumin-corrected serum calcium	14.8 mg/dL (reference range: 8.8-10.8 mg/dL)	Severe hypercalcemia
	PTH	5.2 pg/mL (reference range: 15-65 pg/mL)	Suppressed, supporting PTH-independent hypercalcemia
	25-Hydroxyvitamin D	15.1 ng/mL (reference range: 7.0-53.2 ng/mL)	Not elevated; vitamin D intoxication unlikely
	1,25-Dihydroxyvitamin D	156 pg/mL (reference range: 19.90-79.30 pg/mL)	Elevated; compatible with extrarenal calcitriol production but not disease-specific
	Serum phosphate	3.2 mg/dL (reference range: 2.5-4.9 mg/dL)	Does not support PTH-mediated hypercalcemia
	24-hour urinary calcium	787.5 mg/24 h (reference range: 100-300 mg/24 h)	Marked hypercalciuria
Renal assessment	Serum creatinine	2.77 mg/dL (baseline: 1.01 mg/dL)	Acute kidney injury
	24-Hour proteinuria	1.9 g/day (reference range: <300 mg/day)	Subnephrotic-range proteinuria
	Urinary sediment	Inactive sediment	No evidence of active glomerulonephritis
	Renal ultrasound	Normal	No obstructive or structural cause identified on ultrasound
	Renal biopsy	Chronic tubulointerstitial nephritis	Nonspecific; compatible with, but not diagnostic of, sarcoid-related renal involvement
Malignancy exclusion	Serum and urine immunofixation	Negative	No monoclonal protein identified
	Serum and urine free light chains	Normal kappa/lambda ratios	No evidence of a monoclonal light-chain process
	Bence Jones protein	Negative	Plasma cell dyscrasia less likely
	PTH-related protein	1.10 pmol/L (reference range: <1.50 pmol/L)	Malignancy-related hypercalcemia less likely
	CT of the chest, abdomen, and pelvis	No solid mass	No solid malignancy identified on imaging
Infectious and autoimmune evaluation	Tuberculosis and fungal studies	IGRA, direct mycobacterial examinations, mycobacterial cultures, and fungal cultures were negative	No evidence of mycobacterial or fungal infection in the tests performed
	Viral serologies	HIV, HBV, and HCV nonreactive	No serological evidence of these viral infections
	Autoimmune testing	ANA 1:160; ANCA, anti-dsDNA, anti-SSA/Ro, anti-SSB/La, anti-Scl-70, anti-CCP, rheumatoid factor, and cryoglobulins negative	ANA positive at 1:160, but no specific serological evidence of Sjögren syndrome or another tested systemic autoimmune disorder
Granulomatous disease evaluation	ACE	144 U/L (reference range: 20-70 U/L)	Elevated; supportive but not diagnostic of sarcoidosis
	18F-FDG PET/CT	Hypermetabolic hilar and mediastinal lymph nodes, pulmonary uptake, and salivary gland uptake	Metabolically active abnormalities compatible with inflammatory or granulomatous involvement in the appropriate clinical context
	EBUS-TBNA	Non-necrotizing granulomas	Histological evidence of non-necrotizing granulomatous inflammation; supportive of sarcoidosis after exclusion of alternative causes
	BAL	Lymphocytes: 47.6%; CD4/CD8 ratio: 5.7	Supportive but not diagnostic of sarcoidosis

The differential diagnosis included malignancy-associated hypercalcemia, lymphoproliferative disorders, particularly lymphoma, plasma cell dyscrasia, infectious granulomatous diseases, and systemic autoimmune conditions. The diagnostic workup found no evidence of a solid malignancy or plasma cell dyscrasia. The absence of supporting clinical findings and the results of microbiological, virological, and immunological investigations made infectious and systemic autoimmune etiologies, including tuberculosis and Sjögren syndrome, unlikely (Table 1).

Because of the clinically evident bilateral submandibular gland enlargement, salivary gland USG was performed at admission. It confirmed enlargement of both submandibular glands, measuring 46 mm on the right and 44 mm on the left, with heterogeneous echotexture and hypoechoic areas but without ductal ectasia. The parotid glands were also heterogeneous, without focal nodules or ductal ectasia, and no cervical lymphadenopathy was identified. The patient was subsequently evaluated by the Oral Medicine and General Surgery teams. However, as the glandular enlargement progressively improved following initial treatment, both teams considered that a gland biopsy was unlikely to provide sufficient additional diagnostic benefit and recommended clinical observation.

**Figure 1:**
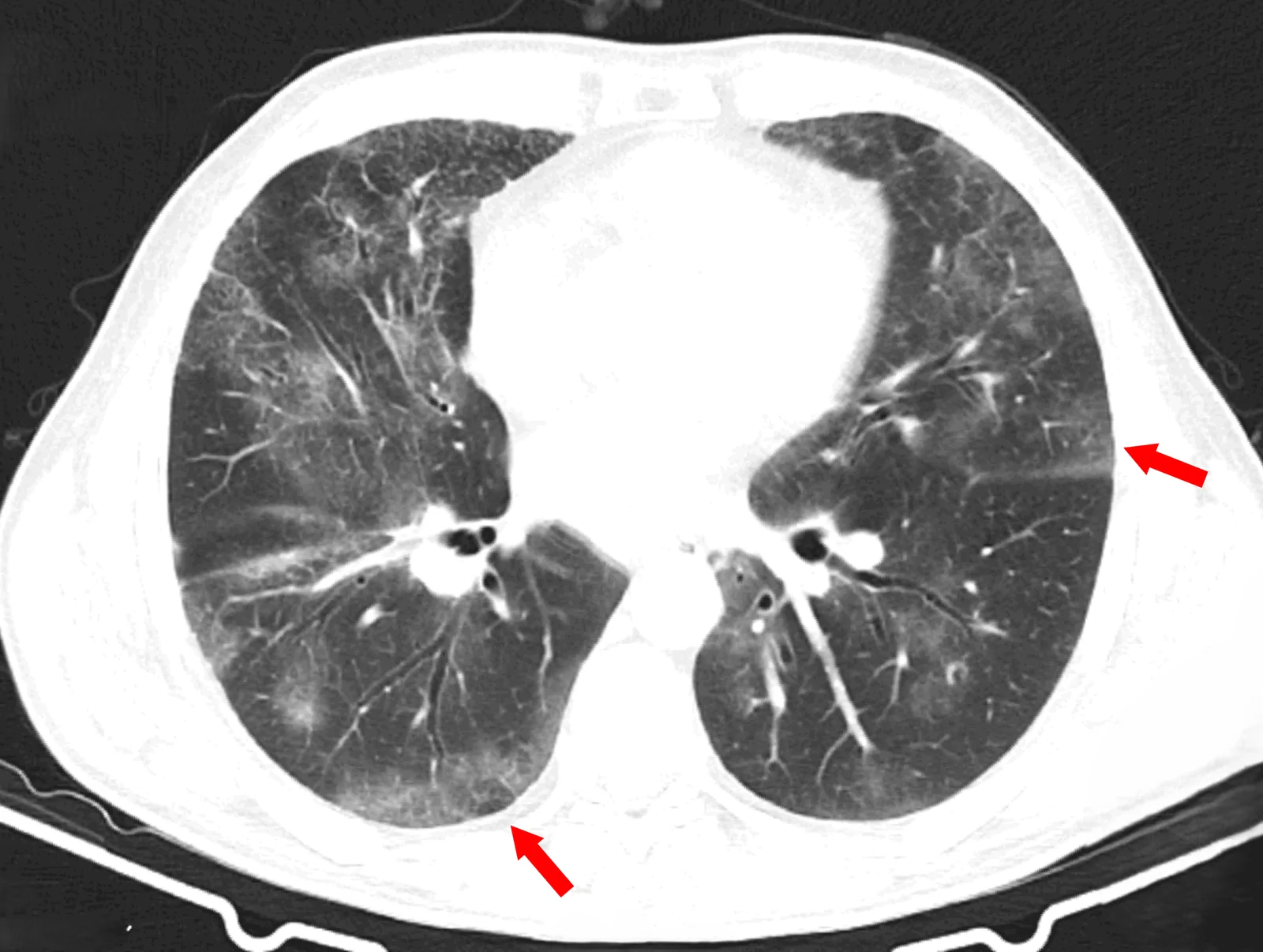
Axial CT image of the chest showing multifocal bilateral ground-glass opacities with a perilymphatic distribution (arrows).

As part of the diagnostic workup, CT of the chest, abdomen, and pelvis was performed. It demonstrated diffuse bilateral pulmonary ground-glass opacities with a perilymphatic distribution, together with mediastinal and bilateral hilar lymphadenopathy (Figure 1). These findings raised suspicion of a systemic granulomatous disease and prompted further etiological investigation.

**Figure 2:**
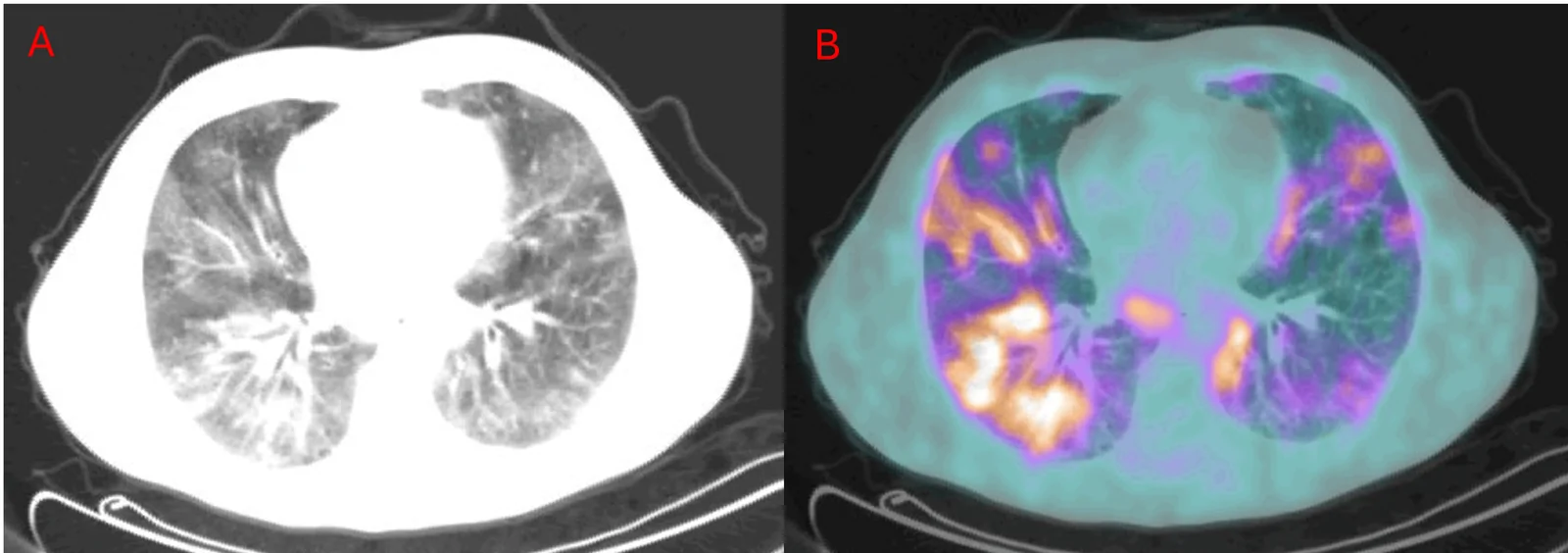
Thoracic findings on 18F-fluorodeoxyglucose PET/CT. (A) Axial CT image showing bilateral pulmonary ground-glass opacities. (B) Fused axial ^18^F-FDG PET/CT image demonstrating increased metabolic activity in the pulmonary parenchyma and hilar regions.

**Figure 3:**
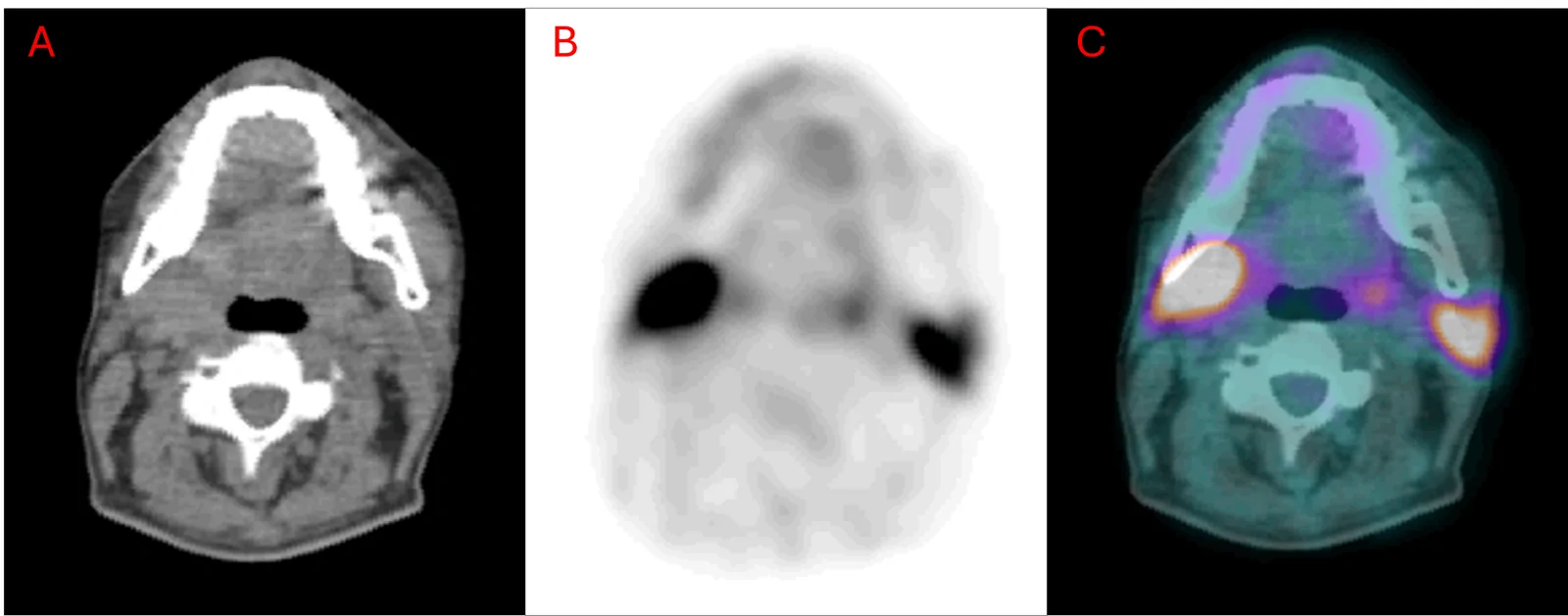
Salivary gland findings on 18F-fluorodeoxyglucose PET/CT. (A) Axial CT image at the level of the salivary glands. (B) Corresponding axial grayscale PET image. (C) Fused axial PET/CT image showing increased ^18^F-FDG uptake in the submandibular and parotid glands, compatible with inflammatory salivary gland involvement.

Further diagnostic evaluation with ^18^F-FDG PET/CT was performed to assess the distribution of metabolically active abnormalities and identify a suitable biopsy target. It demonstrated hypermetabolic bilateral hilar and mediastinal lymph nodes and diffuse pulmonary uptake (Figure 2), as well as increased uptake in the submandibular and parotid glands (Figure 3). These findings were compatible with an inflammatory or granulomatous process, although they were not specific for sarcoidosis.

As histological confirmation was considered necessary, endobronchial ultrasound-guided transbronchial needle aspiration (EBUS-TBNA) was selected as the most appropriate biopsy approach. By then, the salivary manifestations were already regressing under clinical observation, whereas the persistent mediastinal and hilar lymphadenopathy provided a well-defined target for minimally invasive tissue sampling.

**Figure 4:**
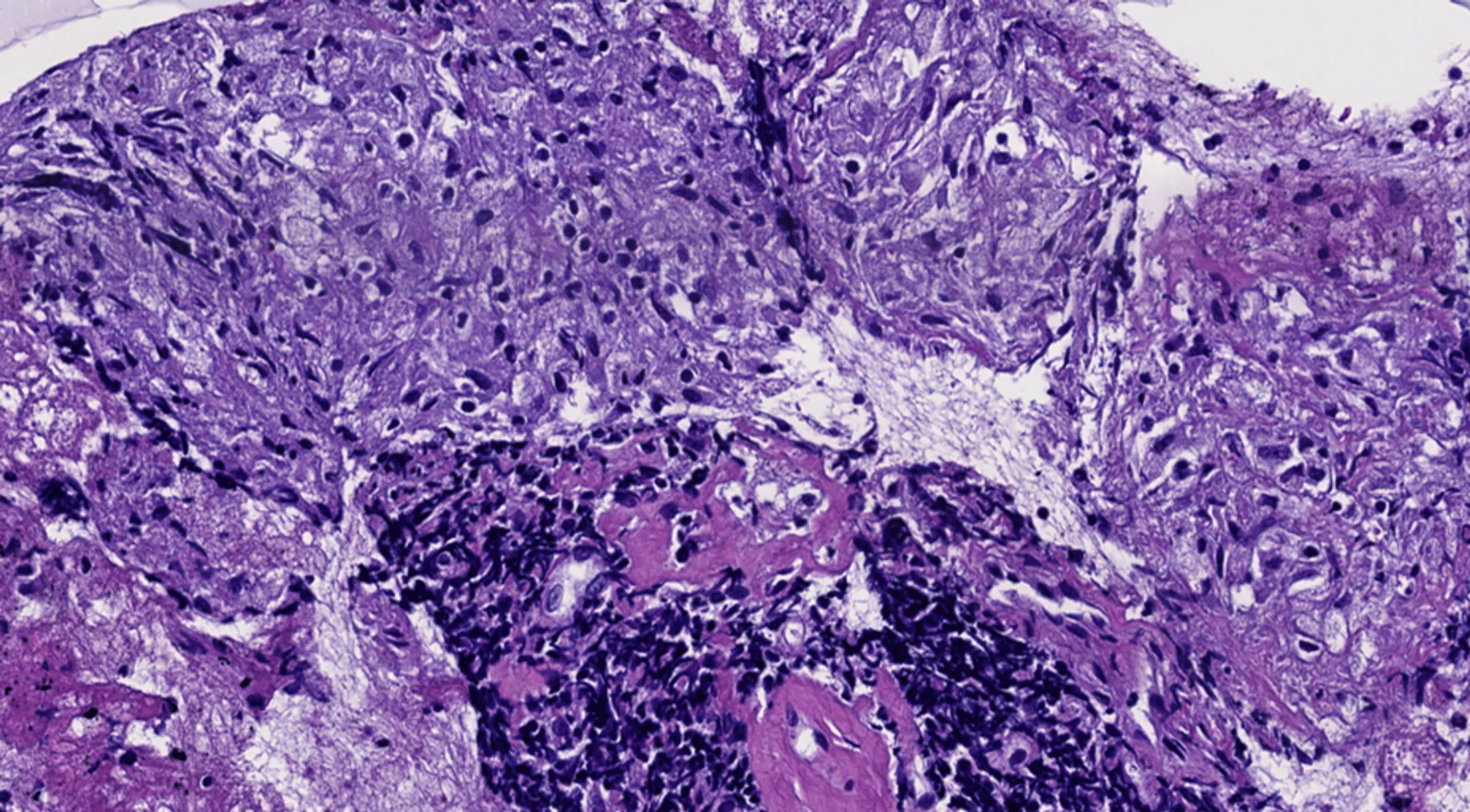
Mediastinal lymph node biopsy findings. H&E-stained section from a station 7 lymph node specimen showing non-necrotizing epithelioid granulomas. Ziehl–Neelsen staining was negative for acid-fast bacilli, and direct mycobacterial examinations and cultures were also negative, providing no evidence of mycobacterial infection in the tests performed.

EBUS-TBNA of a mediastinal lymph node showed non-necrotizing granulomas, without evidence of malignancy or infectious organisms (Figure 4). Bronchoalveolar lavage (BAL) demonstrated lymphocytosis (47.6%) and an elevated CD4/CD8 ratio of 5.7. Although the BAL findings were not diagnostic in isolation, their combination with compatible thoracic imaging, mediastinal lymph node histology, and a negative microbiological evaluation supported the diagnosis of pulmonary sarcoidosis after reasonable exclusion of alternative causes of granulomatous inflammation.

**Figure 5:**
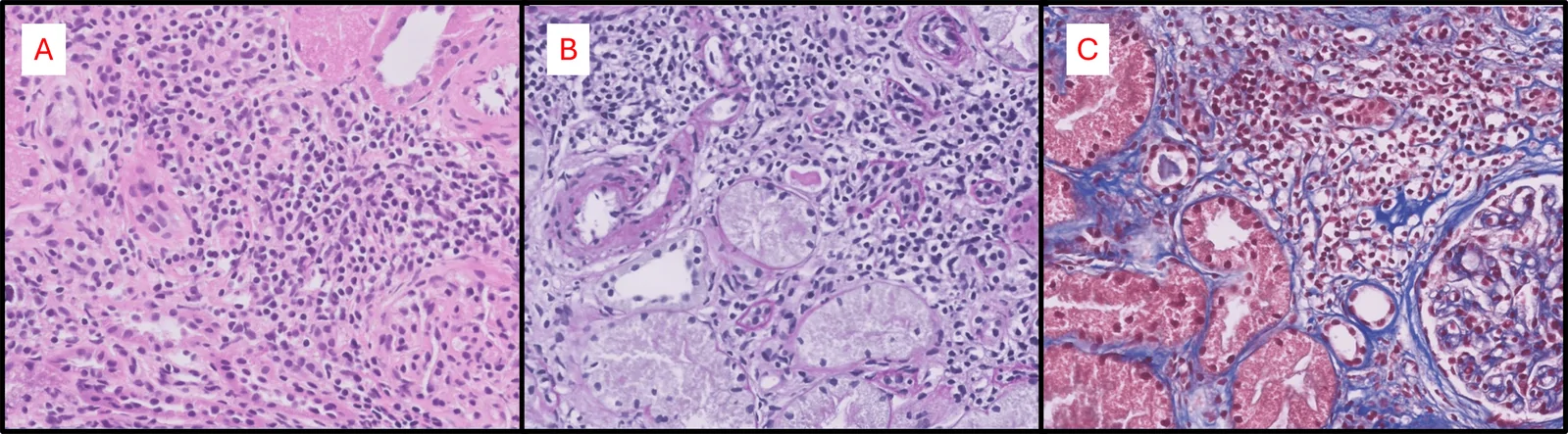
Renal biopsy findings. (A) H&E-stained section showing an interstitial lymphocytic inflammatory infiltrate. (B) Periodic acid-Schiff-stained section showing tubulointerstitial inflammatory changes. (C) Trichrome-stained section highlighting interstitial fibrosis and tubular atrophy. No granulomas were identified in the renal biopsy specimen.

Because renal dysfunction and proteinuria persisted after correction of the hypercalcemia, a nephrology consultation was obtained, and a left renal biopsy was performed electively. It showed chronic tubulointerstitial nephritis with interstitial fibrosis and tubular atrophy but without granulomas (Figure 5). These findings were compatible with, but did not histologically confirm, sarcoid-related renal involvement.

Overall, mediastinal lymph node histology and compatible thoracic imaging supported the diagnosis of pulmonary sarcoidosis. Renal dysfunction was considered multifactorial, reflecting calcium-mediated acute kidney injury with possible concomitant sarcoid-related tubulointerstitial involvement. Salivary gland involvement remained clinically and radiologically suspected but was not histologically confirmed. These findings supported pulmonary sarcoidosis with probable renal and salivary gland involvement.

During the initial hospitalization, severe hypercalcemia was treated with intensive intravenous hydration, followed by intravenous pamidronate and adjunctive furosemide, with close monitoring of urine output. This resulted in normalization of the serum calcium level and an initial improvement in kidney function. Before corticosteroid therapy, the calcium level remained within the normal range, while renal function, proteinuria, and thoracic imaging findings showed further partial improvement. However, kidney dysfunction and proteinuria persisted, as did the mediastinal lymphadenopathy.

Following completion of the diagnostic workup and histological confirmation of thoracic sarcoidosis, oral prednisolone was initiated at 40 mg/day approximately six months after presentation. This dose was maintained for one month and then tapered by 5 mg/month. Corticosteroid therapy was followed by further improvement in kidney function and proteinuria, together with marked regression of the mediastinal lymphadenopathy and bilateral pulmonary ground-glass opacities. This clinical, biochemical, and radiological response was sustained during corticosteroid tapering. The patient continues to receive multidisciplinary follow-up from the internal medicine, nephrology, and pulmonology teams. The main diagnostic milestones, treatment interventions, and objective outcomes are summarized in Table 2.

**Table 2:** Diagnostic timeline, treatment, and clinical course. ^18^F-FDG PET/CT: ^18^F-fluorodeoxyglucose positron emission tomography/computed tomography; BAL: Bronchoalveolar lavage; EBUS-TBNA: Endobronchial ultrasound-guided transbronchial needle aspiration; PTH: Parathyroid hormone.

Time from presentation	Diagnostic or therapeutic milestone	Main findings and clinical course
Month 0 (presentation)	Initial assessment and CT of the chest, abdomen, and pelvis	Severe PTH-independent hypercalcemia (14.8 mg/dL), acute kidney injury (serum creatinine: 2.77 mg/dL; baseline: 1.01 mg/dL), and 24-hour proteinuria of 1.9 g/day. CT showed bilateral pulmonary ground-glass opacities with a perilymphatic distribution and mediastinal and hilar lymphadenopathy.
Initial hospitalization	IV hydration, IV pamidronate, and adjunctive furosemide	At discharge, the serum calcium level had decreased to 10.4 mg/dL and the serum creatinine level to 2.53 mg/dL.
Month 4	Renal biopsy and reassessment	Renal biopsy showed chronic tubulointerstitial nephritis without granulomas. The calcium level remained normal; serum creatinine was 2.37 mg/dL, and proteinuria was 1.0 g/day. CT showed partial spontaneous pulmonary improvement but persistent mediastinal nodes.
Month 5	^18^F-FDG PET/CT	Hypermetabolic hilar and mediastinal lymph nodes, diffuse pulmonary uptake, and increased uptake in the submandibular and parotid glands.
	EBUS-TBNA and BAL	Mediastinal lymph node sampling showed non-necrotizing granulomas. BAL showed lymphocytosis (47.6%) and a CD4/CD8 ratio of 5.7.
Month 6	Corticosteroid therapy	Prednisolone 40 mg/day was started for one month, followed by tapering by 5 mg/month.
Month 8	Early follow-up	Serum creatinine decreased to 1.8 mg/dL and proteinuria to 0.7 g/day; the calcium level remained normal. Chest CT showed marked regression of mediastinal lymphadenopathy and pulmonary ground-glass opacities.
Month 20	Long-term follow-up	Creatinine further improved to 1.4 mg/dL, with sustained clinical, biochemical, and radiological improvement during corticosteroid tapering.

## Discussion

In this case, severe hypercalcemia, renal dysfunction, weight loss, and subsequently identified thoracic lymphadenopathy raised concern for malignancy, primary disorders of calcium metabolism, and granulomatous disease. Hypercalcemia is a recognized complication of sarcoidosis, occurring in approximately 5-10% of patients [2,4-6]. The markedly elevated 1,25-dihydroxyvitamin D level observed in this patient was consistent with dysregulated extrarenal activation of vitamin D by macrophages within granulomatous tissue, leading to increased intestinal calcium absorption and urinary calcium excretion [2,4,5]. Hypercalcemia may therefore represent an early clue to sarcoidosis, but it is neither novel nor specific and often prompts investigation for malignancy and other causes of PTH-independent hypercalcemia [4,5,7,8].

In our patient, the structured etiological workup did not identify evidence of a solid malignancy, lymphoproliferative disorder, plasma cell dyscrasia, or the tested infectious causes, thereby increasing the probability of a noninfectious granulomatous disorder. Negative anti-SSA/Ro and anti-SSB/La test results also reduced the likelihood of Sjögren syndrome. Similar cases have described sarcoidosis presenting with severe hypercalcemia and renal dysfunction, sometimes associated with nonspecific systemic symptoms or neuropsychiatric manifestations [7,8,11-13].

Renal involvement in sarcoidosis can be severe, affecting up to one-third of patients, and may result from both metabolic and inflammatory mechanisms [2,6]. Hypercalcemia and hypercalciuria can contribute to kidney injury through volume depletion, nephrocalcinosis, renal vasoconstriction, and tubular dysfunction, while granulomatous interstitial nephritis reflects direct renal involvement [6,13]. In this patient, both marked hypercalcemia and hypercalciuria were likely contributors to renal dysfunction. Renal biopsy demonstrated chronic tubulointerstitial nephritis but no granulomas, supporting possible renal involvement in the overall clinical context, although it did not provide definitive histological evidence of renal sarcoidosis. Nevertheless, the absence of granulomas does not exclude renal sarcoidosis because granulomatous lesions may be focal, sparse, or missed because of sampling variability [6]. In this setting, the biopsy remained clinically informative, particularly given the persistence of renal dysfunction and proteinuria despite correction of the hypercalcemia.

Pulmonary involvement remains central to the diagnosis and staging of sarcoidosis [1-3,14]. In this case, chest CT demonstrated bilateral mediastinal and hilar lymphadenopathy associated with diffuse perilymphatic ground-glass opacities, corresponding to the lymph node and parenchymal involvement characteristic of Scadding stage II pulmonary sarcoidosis [1-3]. The non-necrotizing granulomas identified in the station 7 lymph node, interpreted together with compatible imaging and the negative microbiological evaluation, provided the strongest evidence for sarcoidosis in this case [3].

^18^F-FDG PET/CT provided an additional functional assessment of disease distribution, demonstrating metabolically active thoracic disease with bilateral hypermetabolic mediastinal and hilar lymph nodes and diffuse pulmonary uptake, as well as increased uptake in the submandibular and parotid glands. Although PET/CT is not routinely required for diagnosis and FDG uptake cannot distinguish sarcoidosis from malignancy, infection, or other inflammatory processes, it can be useful in complex presentations by identifying sites of possible multisystem involvement and helping to select biopsy targets [1,3]. In this case, salivary gland uptake correlated with the patient’s initial clinical presentation of bilateral submandibular enlargement and suggested possible inflammatory involvement, but it did not establish the diagnosis of salivary gland sarcoidosis.

Salivary gland involvement is an uncommon manifestation of systemic sarcoidosis [9,10]. The parotid gland is most frequently affected, often presenting with painless bilateral swelling and occasionally constituting the first manifestation of the disease [2]. Submandibular involvement is less common but should be considered in the differential diagnosis, particularly in female patients [9]. In this case, the clinical swelling, heterogeneous glandular echotexture, and FDG uptake raised suspicion for inflammatory, infectious, or neoplastic etiologies. However, the glands were not biopsied, and the swelling improved spontaneously; direct sarcoid involvement therefore remained suspected rather than confirmed.

Histological confirmation remains essential in most cases, particularly in atypical presentations [2,3]. EBUS-TBNA is recommended as the initial diagnostic procedure for thoracic nodal disease [1-3]. In our patient, it was selected because the salivary gland swelling was improving, USG showed no focal lesion suitable for biopsy, and the persistent mediastinal lymph nodes offered a well-established, minimally invasive target. EBUS-TBNA of a mediastinal lymph node demonstrated non-necrotizing granulomas without evidence of malignancy or infectious organisms, supporting the diagnosis of sarcoidosis in conjunction with compatible imaging and the exclusion of alternative granulomatous disorders [1-3]. BAL provided additional supportive evidence, showing lymphocytosis (47.6%) and a CD4/CD8 ratio of 5.7. Although BAL findings are not diagnostic in isolation, lymphocytosis and a CD4/CD8 ratio greater than 4 significantly increase the diagnostic probability of sarcoidosis when interpreted alongside compatible imaging and histology [1-3].

Serum angiotensin-converting enzyme levels are frequently elevated in sarcoidosis, particularly in active disease, but their diagnostic performance is limited by low specificity and positive predictive value [1,2,15]. In this case, the elevated ACE level provided supportive, although not definitive, evidence for the diagnosis.

Management addressed both the acute metabolic disturbance and the underlying granulomatous inflammatory disease. Initial treatment focused on correcting severe hypercalcemia with intensive IV hydration. Following consultation with the nephrology team, IV pamidronate and adjunctive loop diuretic therapy were administered after volume repletion, with close monitoring of urine output, to enhance calciuresis. The serum calcium level normalized following this initial treatment and remained within the normal range before corticosteroid initiation. During the subsequent diagnostic workup, renal function improved, proteinuria decreased, and follow-up chest CT showed partial spontaneous improvement of the pulmonary abnormalities, although mediastinal lymphadenopathy persisted.

After completion of the diagnostic workup and histological confirmation of sarcoidosis, prednisolone was initiated several months after the initial presentation as first-line treatment for clinically significant pulmonary sarcoidosis and suspected renal involvement [1,3,6,16]. Corticosteroids also reduce macrophage-mediated 1α-hydroxylase activity, thereby decreasing calcitriol production, helping to normalize calcium metabolism, and preventing the recurrence of sarcoidosis-related hypercalcemia [2,4-6]. Further improvement in renal function and proteinuria was observed, and follow-up imaging demonstrated marked regression of the mediastinal lymphadenopathy and pulmonary infiltrates, consistent with a favorable treatment response [16].

This case has some limitations. Although mediastinal lymph node histology supported pulmonary sarcoidosis, the renal biopsy did not show granulomas and therefore did not confirm direct renal sarcoidosis. Renal involvement was therefore inferred from clinicopathological correlation rather than definitive histological evidence. Salivary gland biopsy was not pursued because the swelling was already regressing, and evaluations by the oral medicine and surgical teams supported a conservative approach. Salivary gland involvement therefore remained a clinical suspicion without histological confirmation. Finally, as this is a single case report, the findings cannot establish causality or be broadly generalized.

## Conclusions

This case highlights an uncommon and diagnostically challenging presentation of sarcoidosis, with severe PTH-independent hypercalcemia mediated by calcitriol, renal dysfunction, thoracic lymphadenopathy, and bilateral submandibular gland swelling. Its educational value lies not only in the atypical clinical presentation but also in the stepwise diagnostic workup required to exclude alternative causes of PTH-independent hypercalcemia and establish the diagnosis. This case also emphasizes the importance of integrating biochemical, radiological, and histological findings when organ involvement cannot be definitively demonstrated by a single diagnostic test. The combined evaluation of biochemical findings, serial imaging, renal histology, and EBUS-TBNA findings provides a practical example of how sarcoidosis may be approached when organ involvement is clinically suspected but not uniformly confirmed histologically. Thoracic sarcoidosis was histologically confirmed, whereas direct renal and salivary gland involvement remained clinically suspected but unconfirmed. The renal dysfunction was likely multifactorial, reflecting calcium-mediated injury with possible concomitant tubulointerstitial involvement. Timely recognition, appropriate histological evaluation, and treatment may facilitate metabolic control and renal recovery while reducing the risk of irreversible organ damage. As this report describes a single patient, these observations should be interpreted cautiously, and further studies are needed to better characterize the clinical significance and management of similar atypical presentations.
